# LRSAM1 and the RING domain: Charcot–Marie–Tooth disease and beyond

**DOI:** 10.1186/s13023-020-01654-8

**Published:** 2021-02-10

**Authors:** Paulius Palaima, José Berciano, Kristien Peeters, Albena Jordanova

**Affiliations:** 1 Molecular Neurogenomics Group, VIB-UAntwerp Center for Molecular Neurology, University of Antwerp, Antwerp, Belgium; 2 Service of Neurology, Hospital Universitario Marqués de Valdecilla, Instituto de Investigación Marqués de Valdecilla (IDIVAL), Universidad de Cantabria (UC), Santander, Spain; 3 Centro de Investigación Biomédica en Red de Enfermedades Neurodegenerativas (CIBERNED), Santander, Spain; 4 Professor Emeritus, Department of Medicine and Psychiatry, ‘‘Edificio Escuela Universitaria de Enfermería (Cuarta Planta)’’, University of Cantabria, Avda. de Valdecilla s/n, Santander, Spain; 5 Department of Medical Chemistry and Biochemistry, Medical University-Sofia, Sofia, Bulgaria

**Keywords:** LRSAM1, CMT, Ubiquitin ligase, Peripheral neuropathy, Charcot–Marie–Tooth disease, CMT

## Abstract

In the past decade, mutations in *LRSAM1* were identified as the genetic cause of both dominant and recessive forms of axonal CMT type 2P (CMT2P). Despite demonstrating different inheritance patterns, dominant CMT2P is usually characterized by relatively mild, slowly progressive axonal neuropathy, mainly involving lower limbs, with age of onset between the second and fifth decades of life. Asymptomatic individuals were identified in several pedigrees exemplifying the strong phenotypic variability of these patients requiring serial clinical evaluation to establish correct diagnosis; in this respect, magnetic resonance imaging of lower-limb musculature showing fatty atrophy might be helpful in detecting subclinical gene mutation carriers. LRSAM1 is a universally expressed RING-type E3 ubiquitin protein ligase catalysing the final step in the ubiquitination cascade. Strikingly, TSG101 remains the only known ubiquitination target hampering our mechanistic understanding of the role of LRSAM1 in the cell. The recessive CMT mutations lead to complete loss of LRSAM1, contrary to the heterozygous dominant variants. These tightly cluster in the C-terminal RING domain highlighting its importance in governing the CMT disease. The domain is crucial for the ubiquitination function of LRSAM1 and CMT mutations disrupt its function, however it remains unknown how this leads to the peripheral neuropathy. Additionally, recent studies have linked *LRSAM1* with other neurodegenerative diseases of peripheral and central nervous systems. In this review we share our experience with the challenging clinical diagnosis of CMT2P and summarize the mechanistic insights about the LRSAM1 dysfunction that might be helpful for the neurodegenerative field at large.

## Background

Charcot–Marie–Tooth disease is a progressive and incurable inherited peripheral neuropathy well known for its genetic and phenotypic heterogeneity. The prevalence of CMT is estimated to be between 9.7 and 82.3/100,000 individuals depending on the population, making it the most common disease of its kind [1]. Currently, mutations in over 80 functionally diverse genes were found to be disease-causing with all forms of inheritance observed [2]. Three main clinical types are recognized based on histology and electrophysiology of the median motor nerve. Demyelinating CMT (CMT1) is characterised by nerve conduction velocities < 38 m/s while axonal CMT (CMT2) patients show NCVs > 38 m/s [3, 4]. Finally, a third type with NCVs between 25 and 45 m/s is classified as intermediate CMT (I-CMT) [5].

In this review, we focus on a subtype of axonal CMT, type 2P (CMT2P) that is caused by mutations in the RING-type E3 ubiquitin ligase LRSAM1 (leucine rich and sterile alpha motif containing) [6–11]. *LRSAM1* is associated with both dominant and recessive forms of CMT inheritance.

## Epidemiology

*LRSAM1* mutation carriers were identified in North-America, Europe, and Asia showing a worldwide prevalence [6–8, 10–12]. Despite this, *LRSAM1* is a rare cause of CMT2; it is estimated to account for < 1% of all CMT2 cases as tested in German, Dutch, and Sardinian populations [7, 13, 14]. The prevalence rate could still be an underestimation as the mild phenotype and reduced penetrance makes it difficult to identify the mutation carriers [10]. This is illustrated by the presence of known dominant disease-causing mutations (p.Cys694Arg; p.Glu674ArgfsTer83) in the genome aggregation database (gnomAD) containing over 123,136 whole exome sequences and 15,496 whole genome sequences collected from individuals with no known severe paediatric illness [7, 15–17].

**Fig. 1:**
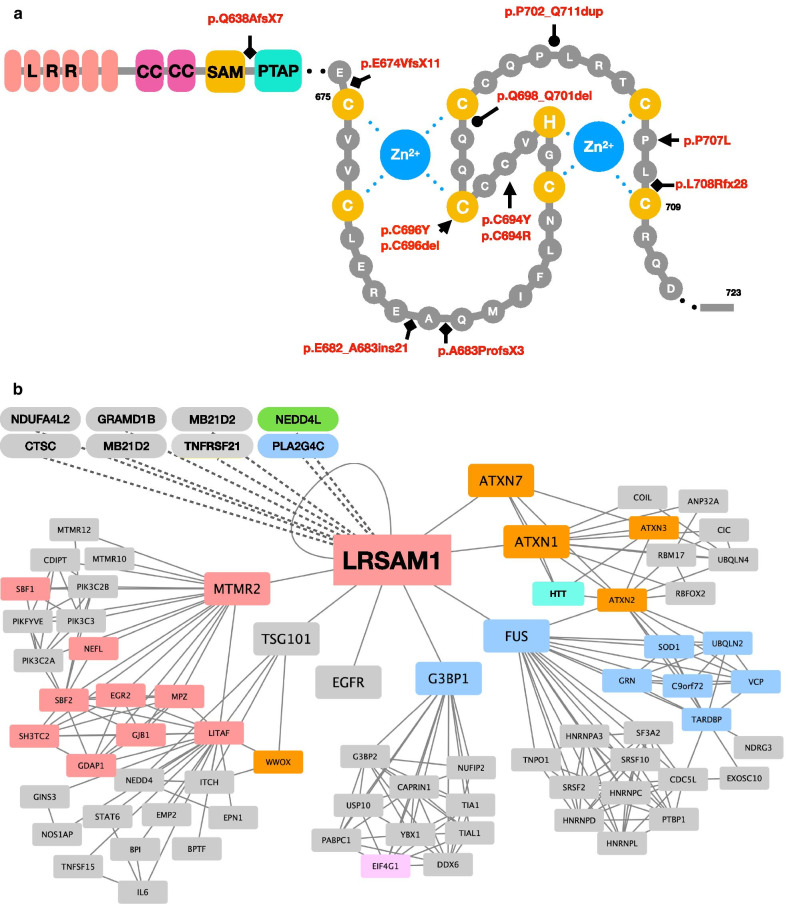
**a** The domains and location of CMT-causing mutations in LRSAM1. LRR, leucine rich repeat; CC, coiled-coil; SAM, sterile alpha motif; PTAP, Pro-Thr-Ala-Pro motif; RING, really interesting new gene (expanded view). Frameshift mutations are indicated by square indentations, missense mutations by arrows, in-frame changes—circle indentations. Critical residues required for the binding of zinc ions (blue) are indicated in yellow. **b** LRSAM1 interaction network obtained from STRING using 10 interactors in the first shell and high confidence (0.7). The individual networks were merged using Cytoscape. Squares—proteins, ovals—affected transcripts. Solid lines indicate direct protein–protein interactions. Dashed lines correspond to data obtained from RNAseq experiments. Colours indicated genes with known disease-causing mutations: grey—no known mutations; pale red—CMT; orange—spinocerebellar ataxia; blue—ALS; pink—Alzheimer’s disease; green—Parkinson’s disease; yellow—Huntington’s disease

A total of 12 CMT-causing mutations were discovered in *LRSAM1* (Fig. 1a). The c.1913-1G > A splice site mutation (p.Glu638AlafsX7) was the first one described and remains the only known cause of recessive CMT2P [6]. It is positioned outside of known functional domains [6]. The rest of the known mutations are dominantly inherited and are located in the catalytic C-terminal RING domain of LRSAM1. Their tight clustering suggests that the RING domain is a mutation hotspot for CMT2P (Fig. 1a). The majority of the dominant CMT2P mutations can be attributed to a single individual or pedigree. An exception to this is a recently identified p.Gln698_Gln701del found in seven French pedigrees. Subsequent haplotyping using WES identified a 100–450 kb region surrounding the mutation shared by five of those families indicating a founder effect [18]. The heterozygous p.Ala683ProfsX3 identified in two unrelated patients of Sardinian descent could be a founder mutation too [9, 13]. Indeed, this island population was effectively isolated from the mainland Europe ~ 140–250 generations ago [19] and screenings in other Mediterranean populations failed to identify mutations in *LRSAM1* [9, 13, 14].

## Clinical features

In dominant CMT2P pedigrees, the disease onset is between 2nd and 6th decades of life (Table 1). Several mutation carriers were described with a very mild phenotype manifesting later in life, highlighting the importance of longitudinal clinical follow-ups in the diagnosis of *LRSAM1* pathology [9, 10]. The magnetic resonance imaging of lower limb musculature was shown to be an essential tool to detect these otherwise asymptomatic individuals [10]. Yet, individuals that remain fully asymptomatic have also been reported making the diagnosis of *LRSAM1*-neuropathy even more problematic [9, 10].

**Table 1:** Clinical data from CMT2 pedigrees associated to LRSAM1 mutations

	Guernsey et al. [6]	Weterman et al. [11] Aerts et al. [23]	Nicolau et al. [9]	Engeholm et al. [12]	Peeters et al. [10] Berciano et al. [21]	Hu et al. [8]	Peddareddigari et al. [51]	Hakonen et al. [7]	Zhao et al. [17]	Peretti et al. [18]	Mortreux et al. [52]
No. of pedigrees	1	1	1	1	1	1	1	1	1	8	1
No. examined patients	6	11	10	3	13	5	3	2	4	72	1
No. asymptomatic patients	1	2	6	None	6	None	2	None	None	1	None
Transmission	AR	AD	AD	AD	AD	AD	AD	AD	AD	AD	AD
Age onset, proband/secondary cases	20/adulthood	32/20–40	34/15–50	20/20–40	17/10–40	30/20–43	10 (proband)	50 (proband)	32 (42–49)	39 ± 12 (15–70)	56
Presenting symptom	Distal leg weakness	Distal leg weakness	Distal leg weakness	Foot hypoesthesia	Foot deformity	Gait clumsiness	Gait clumsiness	?	Distal leg weakness	Gait instability	Food drop
dLL weakness or amyotrophy	+	+	+	+	+	+	+	?	+	+	+
dLL hypoesthesia	+	+	+	+	+	+	+	?	+	+	+
LL reflexes	Absent	Absent or reduced	Absent (Achilles)	Absent	Preserved or absent	Absent (Achilles)	Present or reduced	?	Absent or reduced	Usually absent	Absent
Pes cavus	+	+	+	No	+	No	No	?	+	Usually absent	+
dUL weakness or amyotrophy	+	+ in 3 cases	No	No	No	No	+	?	+	No	No
dUL hypoesthesia	+	+ in 7 cases	No	+	Usually mild deficit	No	+	?	+	Usually present	No
UL reflexes	Absent	Absent or reduced in 7 cases	Preserved	Preserved	Usually preserved	Preserved	Preserved	?	Reduced	Preserved	Preserved
Pain	Episodic cramping	No	Episodic cramps	Cramps	No	No	No	?	Tingling feet	Cramps, neuropathic pain	No
Autonomic dysfunction	Present^a^	No	Present^a^	No	No	No	No	?	No	Present^a^	No
Other manifestations	Sensory ataxia; tremor	Parkinson in 3 cases	Present^c^	Tabetic gait	None	Pelvic swing	None	?	None	Tremor; restless leg syndrome	No
Disease severity	Variable to wheelchair bound	Mild	Asymtomatic to	Mild	Mild	Mild to moderate	Moderate	?	Mild to moderate	Mild to wheelchair bound	Moderate
Serum CK level	↑ 1921	Not done	Not done	↑480	Not done	Not done	Not done	?	Not done	↑ 3000	Not done
Nerve conduction pattern	Axonal	Axonal	Axonal	Axonal (attenuated SNAP)	Axonal	Axonal	Axonal	?	Axonal	Axonal	Not done
Electromyographic pattern	Neurogenic^b^	Not done	Not done	Neurogenic	Neurogenic	Neurogenic	Neurogenic	?	Not done	Neurogenic	Not done
Nerve biopsy pattern	Not done	Axonal	Not done	Axonal	Axonal^d^	Not done	Not done	?	Not done	Axonal	Not done

AD, autosomal dominant; AR, autosomal recessive; CK, creatin kinase; dLL, distal lower limb; dUL, distal upper limb

(a) Erectile dysfunction and/or urinary urgency. (b) Including paraspinal muscles; fasciculations and fibrillations. (c) Mutilating arthropathy; fasciculations. (d) See text for autopsy study in one case, sural nerve biopsy in two cases, and proximal-to-distal histological study of sciatic nerve in an amputated leg; in short, the disease is a model of motor and sensory neuronopathy with length-dependent sensorimotor axonal degeneration. ( +) Present sign

**Fig. 2:**
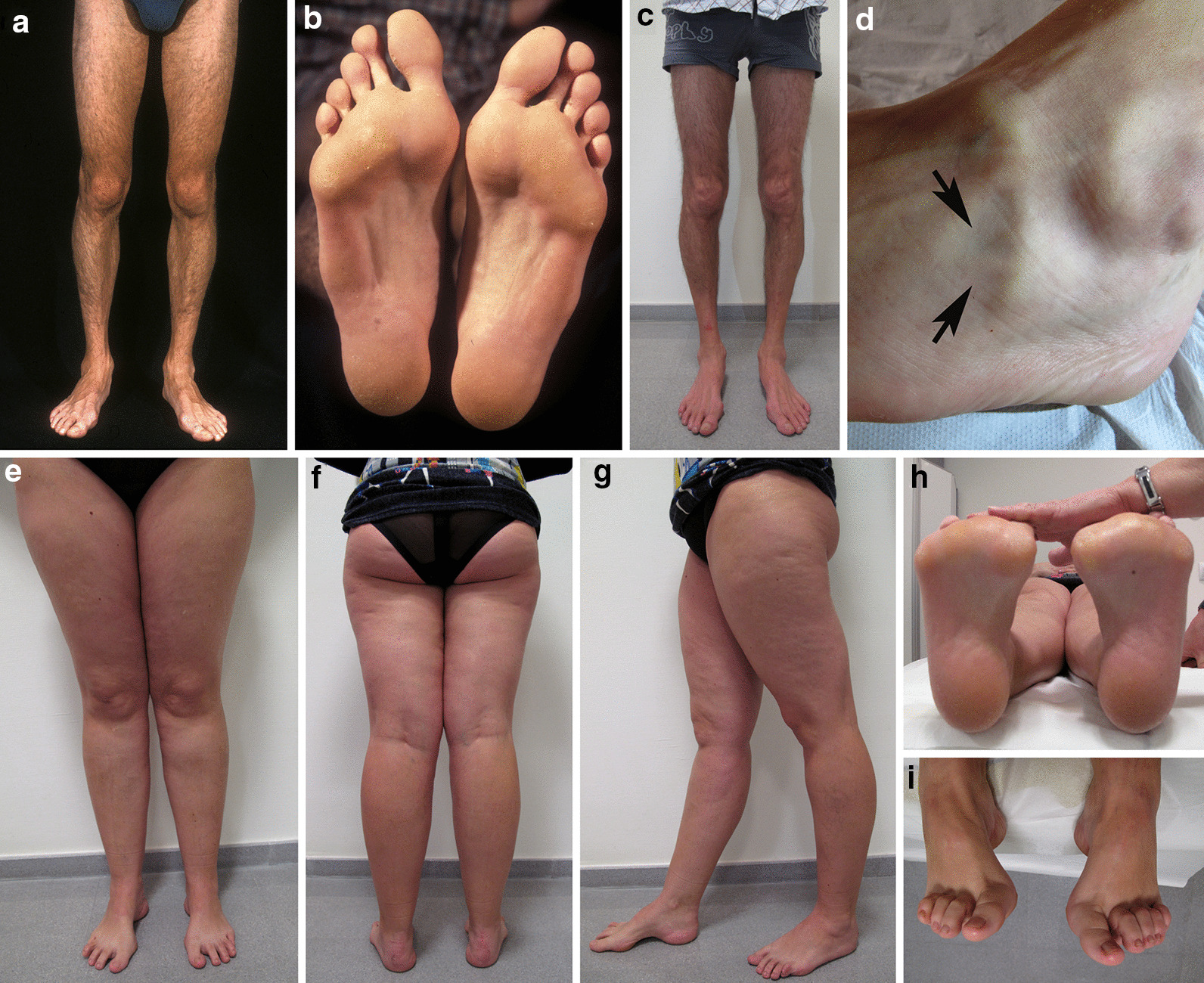
Clinical phenotype. **a**–**d** Serial pictures of Case IV-4, and pictures of Case IV-11 taken at age 32 years **e**–**i** from the pedigree reported by Peeters et al. [10]. **a** At age 25, there is normal appearance of lower limbs; particularly note the absence of peroneal muscular atrophy and toe clawing. **b** Close-up picture of the sole of the feet showing midfoot hollowing and callosity over transverse arcus plantaris and external foot borders. **c** At age 43, note the appearance of lower-leg amyotrophy mainly involving peroneal musculature; in spite of this there was evidence of neither weakness of foot extensors/evertors nor difficulty in heel walking. **d** Close-up picture of the left foot showing wasting of extensor digitorum brevis muscle (arrows). **e**–**g** There is no evidence of lower-leg amyotrophy; note that even under load pes cavus and clawing of toes are visible **e**, **g** and **h**–**i** Close-up pictures of the feet showing marked pes cavus-varus deformity and toe clawing. Reproduced from Peeters et al. [10]

In dominant pedigrees, the usual clinical hallmark is a relatively mild, very slowly progressive axonal neuropathy, mainly involving the lower limbs, with age of onset in the second or third decade of life [11]. Over four decades, one of the authors (JB) has performed serial clinical evaluations in a large CMT2P pedigree comprising 11 *LRSAM1* mutation carriers in three generations, and eight at-risk non-affected subjects [10]. Symptomatic onset occurred between the second and the fifth decade of life in 5 patients, the remaining 6 being asymptomatic. The main presenting symptoms were foot deformity and difficulty in walking; neither sensory nor dysautonomic symptoms did occur. The affected members were at the most only moderately disabled; using the CMT neuropathy scale (CMTNS) [20], the scores ranged between 6 and 11 in all 5 patients aged from 58 to 84 years, and between 0 and 3 in all 6 patients between 35 and 47 years of age. Serial clinical evaluation was crucial not only for detecting disease progression (Fig. 2a–d), but also to establish the affection statuses [10]. Established clinical picture included pes cavus (Fig. 2e–i) in all but one patient, and a variable combination of signs comprising lower-limb areflexia, stocking hypoesthesia, and leg amyotrophy and weakness that was markedly asymmetric in one patient [21]. Upper-limb areflexia and mild glove hypoesthesia were only observed in 3/5 patients older than 50 years. Leaving foot deformities aside, four mutation carriers, aged from 35 to 47 years, had normal examination; they are examples of reduced penetrance, a phenomenon that had been observed in other CMT2P pedigrees [9–11].

Forefoot pes cavus is a cardinal manifestation of any CMT subtype, and as such was observed in 10 of our 11 CMT2P patients [10, 21]. The high prevalence of forefoot pes cavus in CMT2P indicates that denervation of intrinsic foot musculature, which initiates cavus deformity, is an early event [22]. In this regard and when confronted with a sub-clinical patient, MRI of lower-limb musculature can be most helpful in detecting fatty atrophy of foot muscles [10].

In the only AR-CMT2P pedigree, affected individuals showed a clinical picture comparable to that reported in dominant pedigrees, though some of them became wheelchair dependent [6]. Heterozygous *LRSAM1* mutation carriers were asymptomatic.

Other inconstant clinical characteristics of CMT2P are erectile dysfunction, urgency of urination, severe evolution to wheelchair bound, episodic numbness, paresthesias, cramps, absence of foot deformities, trophic disorders of the foot with mutilating arthropathy, predominantly sensory neuropathy with fasciculations and hyper-CK-aemia, and parkinsonism [6, 8, 9, 12, 17, 23]. In the CMT2P pedigree reported by Peretti et al. [18] the clinical picture was dominated by sensory ataxia, neuropathic pain, and length-dependent sensory loss to all modalities.

## Electrophysiological features

Electrophysiological studies usually show the characteristic findings of axonal CMT, namely nerve conduction velocity slowing > 60% of the lower limit of normal [24], though in initial stages of the disease they might be normal. Absence of motor or sensory responses in lower-limb nerves may occur in severe cases. In nerves with severe attenuation of compound muscle action potentials or sensory nerve action potentials, the resulting nerve conduction velocities might be in the intermediate range (30 to 40 m/s for median nerve) [9, 17], a fact indicative of preferential involvement of larger myelinated fibres [25].

Electromyography of distal lower-limb muscles, particularly extensor digitorum brevis and tibialis anterior [10, 18], often reveals a pattern of chronic denervation. Denervation of paraspinal muscles, indicative of involvement of very proximal nerve trunks, has exceptionally been reported [6]. As might have been expected in a chronic disorder, the presence of active denervation potentials (fibrillations or positive waves) is rarely reported [6].

## Imaging features

Peeters et al. [10] have reported the only systematic MRI investigation of lower-limb musculature in CMT2P, comprising 2 symptomatic patients, 5 asymptomatic carriers, and 1 individual not carrying the disease-causing mutation. Only in the subject not carrying the disease-causing mutation was lower-leg and foot musculature preserved [10]. In the other 7 (all mutation carriers), MRI consistently revealed changes, which were more evident with disease progression [10]. There was fatty infiltration in foot and calf musculature with proximal-to-distal gradient of distribution that correlates well with length-dependent axonal degenerations found in pathological studies (see below).

## Pathological studies

**Fig. 3:**
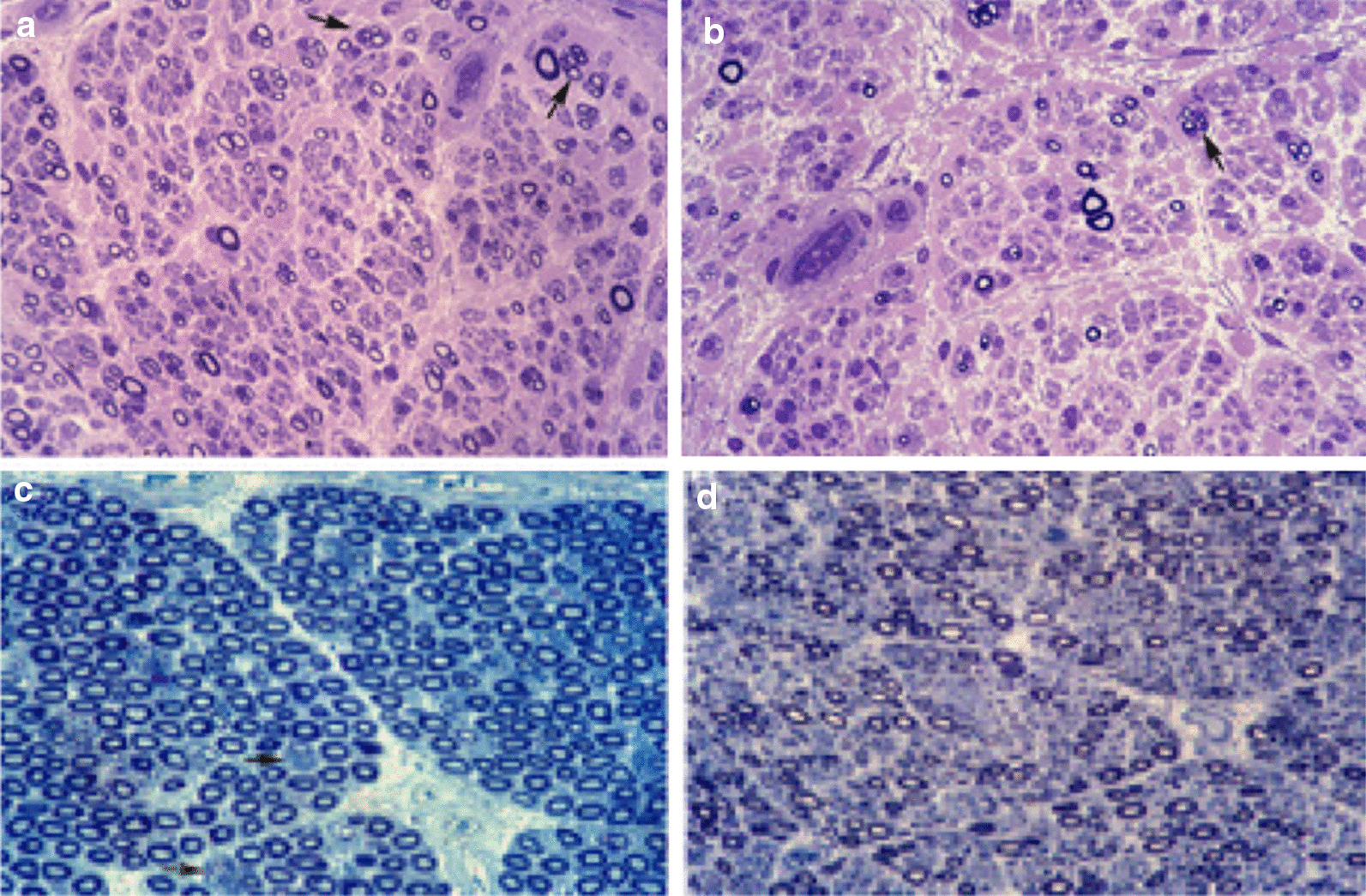
Tibial nerve pathology and necropsy findings. The tibial nerve was dissected down in an amputated leg of Case III-5 in Berciano et al. [21] and Case III-1 in Peeters et al. [10], when the patient was aged 39 years. Semithin transverse sections of the upper third (**a**) and the lower third (**b**) of the nerve showing loss of myelinated fibres, with an evident proximal-to-distal gradient in this fibre loss; note that the density of myelinated fibres is far lower than that of lumbar roots (see below). Several fibres contain thin myelinated sheaths in relation to axon diameter, a feature suggestive of remyelination. Note also clusters of regenerating fibres (arrows) (Toluidine blue, × 250 before reduction). The propositus died at age 32 years (Case III-7 in Berciano et al., [21], and Case III-4 in Peeters et al., [10]). Semithin sections of L5 ventral (**c**) and dorsal (**d**) roots showing that larger fibres are reduced (particularly in dorsal root), but smaller fibres are increased (for mophometry, see Berciano et al., [21]). Note the clusters of regenerating fibres (arrows) (Toluidine blue, × 240 before reduction). Adapted from Berciano et al. [21]

In the CMT2P pedigree reported by Berciano et al. [21] and Peeters et al. [10], extensive pathological investigations were performed, comprising an autopsy study, 2 sural nerve biopsies, and nerve trunk dissection from an amputation leg at the lower third of the thigh where the following nerves were removed: sciatic, tibial, lateral plantar, medial plantar, common peroneal, and the deep peroneal and its lateral terminal branch. Histological study of sural nerves and sciatic nerve and its branches revealed loss of myelinated fibres with a proximal-to-distal gradient of fibre loss, and clusters of small regenerating fibres (Fig. 3a,b) (for morphometry, see Berciano et al. [21]). Similar findings have been reported in biopsies of sural nerve or superficial peroneal nerve of *LRSAM1* carriers [12, 18]. Post mortem study showed loss of anterior horn and dorsal root ganglion neurons in the lumbar and sacral segments, and degeneration of the *fasciculus gracilis* [21] An additional figure shows more details (see Additional file 1: Supplementry figure 1). In L5 ventral and dorsal roots there was a normal number of myelinated fibres and axonal atrophy (Fig. 3c,d), diameter histograms being shifted to the left because of a significant loss of large myelinated fibres and regeneration [21]. These anatomical findings are consistent with a primary neuronopathy affecting motor and sensory neurons with length-dependent sensorimotor axonopathy [21].

## LRSAM1 structure

*LRSAM1* is located on chromosome 9q33.3 and consists of 26 exons spanning a 51 kb genomic region. The gene shows evolutionary conservation in chordates and no ortholog has yet been found in other phyla [26]. Four of the nine known isoforms of *LRSAM1* are protein-coding; they do not show any tissue specificity and are ubiquitously expressed. Intriguingly, *LRSAM1* is highly expressed in human foetal and adult nervous system, and particularly in motoneurons of the spinal cord and sensory ganglia [11].

LRSAM1 is a member of the ubiquitin proteasome system (UPS) which consists of enzymes involved in ubiquitin activation (E1), conjugation (E2) and ligation to target (E3). As a RING-type E3 ligase, it contains a canonical RING-motif (Cys-X2 -Cys-X(9–39) -Cys-X(1–3) -His- X(2–3) -Cys-X2 -Cys-X(4–48) -Cys-X2 –Cys) in which highly conserved cysteines and histidine are separated by variable numbers of other (X) residues (Fig. 1a) [27, 28]. The Cys_(7)_-His lattice binds two zinc ions that are pivotal in maintaining the three-dimensional structure of the domain [27, 28] (Fig. 1a). The RING is essential for the binding of the ubiquitin-conjugating enzymes (E2) and facilitates the direct transfer of the activated ubiquitin to the target proteins by bringing the E2 and the substrate into close proximity [28].

Similar to other RING-type ligases, LRSAM1 forms homodimers both in vitro and in vivo [7, 27–29]. This self-association is critical to the auto-ubiquitination activity that is one of the auto-regulatory mechanisms of the E3 ligases [27]. While the RING domain is pivotal in the ubiquitin transfer, the dimerization is regulated by the CC_1_ (coiled-coil-1) and LRR (leucine-rich repeats) domains [30]. The sterile alpha motif (SAM), and PTAP (Pro-Thr/Ser-Ala-Pro) and the second coiled-coil (CC_2_) domains are responsible for substrate interaction and were observed to impact the ubiquitylation function in vitro [30, 31]. Finally, the six N-terminal LRRs also play a role in pathogen/substrate recognition [32].

## LRSAM1 function

Ubiquitination is a key cellular process that regulates protein activity or marks them for degradation [31, 33]. As an E3 ubiquitin ligase, LRSAM1 is involved in multiple cellular processes from RNA metabolism and transcriptional regulation to endosomal sorting and xenophagy. Even though the protein executes this canonical function in the cytoplasm, it contains a nuclear localization signal and has been found in the nucleus as well [8, 34].

To date, the only identified LRSAM1 ubiquitination target is the tumour suppressor gene 101 (TSG101) [31]. TSG101 is a member of the ESCRT I (endosomal sorting complex required for transport) and is involved in vesicle recycling, retroviral Gag protein assembly and the budding of viral-like particles [31, 35]. The inactivation of TSG101 through ubiquitination by LRSAM1 results in degradation of epidermal growth factor receptor (EGFR) and a reduction in viral budding [26, 31]. This interaction is of particular interest as EGFRs (e.g. Erbb2, Erbb3) are known to be involved in the CMT pathology [36]. Also, TSG101 associates with LITAF (LPS-induced TNF-activating factor), a known CMT1-causing protein but as the CMT2P patients do not show signs of demyelination, the relevance of this interaction is unclear [37].

LRSAM1 is likely involved in the RNA metabolism. An interactomics study performed on *Lrsam1* knockout mouse motoneuron-like cells (NSC34) transfected with human LRSAM1^WT^ or LRSAM1^Cys694Arg^ identified multiple RNA binding proteins [8]. Two of them known to be involved in mRNA splicing (FUS) or poly-A tail preservation (G3BP1) were verified in patient-derived fibroblasts [8, 38, 39].

Lastly, LRSAM1 was implicated in cell adhesion regulation through mediating the levels of beta-catenin and E-cadherin through a GSK-3 dependent mechanism in rat PC12 cells [34]. However, these findings were never pursued further, and their implications are unknown.

## Modelling LRSAM1

To study the loss of *LRSAM1* associated with the recessive CMT mutation, a zebrafish LRSAM1 knockdown and a mouse *Lrsam1* knock-out (*Lrsam1*^*KO*^) model were created [11, 40]. Both organisms carry orthologs in their genome that share 58% and 88% identity to human LRSAM1 respectively (Clustal Omega) [41]. The morpholino knock-down of the zebrafish ortholog induced a strong neurodevelopmental phenotype [11]. The embryos exhibited reduced motor neuron organization, which did not improve at four days post fertilization indicative of the involvement of Lrsam1 in neuronal development [11]. Although a neurodevelopmental phenotype has not been reported in CMT2P patients, the study highlights the importance of *LRSAM1* in the nervous system (Fig. 1b).

The *Lrsam1*^*KO*^ mouse model was developed using gene trap mutagenesis resulting in a complete loss of protein expression (40). Contrary to the findings in zebrafish, *Lrsam1*^*KO*^ mice resembled their wild-type littermates even at 12 months of age [40]. The lack of neurodegenerative phenotypes in murine models has been reported previously for a CMT-causing gene (*HINT1*) [42]. Interestingly, the *Lrsam1*^*KO*^ mice did exhibit stronger signs of axonal degeneration when challenged with acrylamide as a neurotoxic agent [40]. After two weeks of acrylamide treatment, wild type, heterozygous and homozygous mutant mice showed overt signs of neurological dysfunction, such as tremor and poor coordination. Quantitative measures, including NCV studies and axon measurements, demonstrated that the degeneration was more severe in the mutant animals. Upon reduced Lrsam1 expression levels, axons of the motor, but not sensory, branch of the femoral nerve showed greater sensitivity to acrylamide-induced degeneration. In addition, the NCVs measured between the sciatic notch and the lumbrical muscle of the foot was significantly reduced after acrylamide treatment in mutant animals compared to their wild type littermates. Similarly, an exacerbation of the phenotype has been observed in mildly affected patients carrying mutations in *PMP22*, *SH3TC2*, and *MPZ* undergoing chemotherapy, supporting the relevance of the model [43].

## Mutation effect

The only known recessive mutation in LRSAM1 leads to a complete loss of protein as detected in EBV-transformed lymphocytes from a patient [6]. Conversely, LRSAM1 was detected in the dominant mutation carriers and the expression levels were unchanged [9, 10]. The tight clustering of dominant mutations highlights the importance of the RING domain as a driver of the disease. Frameshift mutations cause either premature truncation (p.Glu682_Ala683ins21; p.Ala683ProfsX3; p.Glu674ValfsX11) or elongation (p.Leu708Argfx28) of the RING domain [9, 11, 12]. The missense mutations target highly conserved residues within the RING-motif possibly affecting the binding of the two zinc ions leading to the destabilization of the RING domain (Fig. 1a) [7, 8, 10]. The neurotoxicity of the mutant protein was demonstrated through the expression of the human LRSAM1^Cys694Arg^ in murine Lrsam1^KO^ neuronal cells (NSC34). The lack of *Lrsam1* in these cells did not induce neurodegeneration, corroborating the findings in the mouse model [40], however overexpression of human *LRSAM1*^*Cys694Ar*g^ induced axonal degeneration [8].

Some of the most informative clues on how *LRSAM1* mutations affect its function comes from the study of an artificially created *LRSAM1*^*Cys675Ala*^ mutation [31]. The *LRSAM1*^*Cys675Ala*^ produces a stable protein product but affects a critical residue in the RING domain rendering the protein catalytically dead [7, 31]. Expression of it in HeLa cells containing a LRSAM1^WT^ altered the localization of the retroviral Gag protein and both proteins co-localised to circular cytosolic structures in a dominant negative manner [31]. This was hypothesized to be a consequence of the loss of TSG101 regulation through ubiquitination that disturbed the sorting of cargo vesicles. Supporting this, CMT-mutants (LRSAM1^Cys694Tyr^, LRSAM1^Pro707Leu^, and LRSAM1^Leu708Argfx28^) while retaining their ability to interact with LRSAM1^WT^, lose the capacity to associate with the E2-conjugating enzyme Ubc13 in yeast-2-hybrid experiments [7]. This provides a potential explanation to why they show a significant reduction in their ubiquitination activity [7].

The LRSAM1^Cys694Arg^ mutation also showed significant reduction in the interaction with FUS and G3BP1 [8], two proteins implicated in other neurodegenerative diseases, and to have an effect on their localization. FUS and G3BP1 exhibited reduced expression in the nucleus of patient-derived LRSAM1^Cys694Arg^ fibroblasts and mouse neuronal *Lrsam1*^*KO*^ cells overexpressing the mutant protein [7]. Strikingly, this effect was extended to TDP-43, which does not interact with LRSAM1 directly, but complexes with FUS in the regulation of mRNA processing [8, 44].

A parallel transcriptomics study performed on EBV-transformed lymphocytes derived from patients carrying the *LRSAM1*^*Cys694Tyr*^ mutation provided further insight into the disease mechanism [10]. One of the validated transcripts showing significant upregulation was death receptor 6 (*DR6*, also known as *TNFRSF21)*, a well-studied facilitator of Wallerian degeneration [10, 45]. The data also detected an up-regulation of *NEDD4L* [10]. Unlike LRSAM1, NEDD4L is a HECT-type E3 ubiquitin protein ligase and is known to also target TSG101. Therefore, its upregulation could be partially compensating for the defective LRSAM1 in the ESCRT-I regulating pathway [10].

## LRSAM1 in the broader context of neurodegeneration

Several independent observations provide an attractive setting for studying *LRSAM1* as a possible modifier of different neurodegenerative diseases. For example, four males from two pedigrees suffering from dominant CMT2P were observed to develop Parkinson’s disease [11, 18, 23]. In three related patients carrying the *LRSAM1* p.Leu708Argfx28 mutation genetic causes in the 14 most common PD- causing genes were excluded leaving this variant potentially causatively linked [23]. Moreover, previous transcriptomics analysis performed on patients carrying LRSAM1^Cys694Tyr^ identified a strong upregulation of the ubiquitin ligase *NEDD4L.* Downregulation of this gene was shown to alleviate motor dysfunction in mouse model of PD [46]. These observations provide possible venue to explore the role of LRSAM1 as a causal or risk factor in PD.

In the context of ubiquitination-induced aggregate clearing, LRSAM1 has also been implicated in Huntington’s disease. It was observed that mice expressing Htt^Q300^ exhibited upregulation of Lrsam1 that correlated with increased clearing of htt171-82Q aggregates [47]. This is contrary to the findings published by another group showing that LRSAM1 has no impact on protein aggregate clearing [32].

The neurodevelopmental defect observed in the zebrafish morpholino knock-down of *LRSAM1* is reminiscent of the phenotype of individuals suffering from Hirschsprung's disease (HSCR) [11, 48]. HSCR is a severe congenital disorder caused by an impaired neural crest development, resulting in the absence of intestinal ganglion cells and causing no bowel movements in infants [48]. The miRNA-939 is significantly up-regulated in patients with HSCR when compared to controls, down-regulating *LRSAM1* mRNA and potentially contributing to the disease development in these patients [48]. So far, no HSCR-like phenotype has been described in the recessive CMT2P patients that completely lack *LRSAM1*.

The interaction of LRSAM1 with FUS and the observed effect on the localisation of TDP-43 in mutation carriers is very intriguing. Both proteins are associated with FTD/ALS and exhibit similar nuclear exclusion (Fig. 1b) [49, 50]. However, there is no formation of aggregates of FUS or TDP-43 in the CMT2P patient-derived cells. It is worth exploring whether LRSAM1 could have a modifying effect on patients carrying a pathogenic mutation in FUS or TDP-43 by accentuating the exclusion of those proteins from the nucleus.

A plausible first instance of *LRSAM1* as a CMT modifier gene was the report of a heterozygous missense c.643C > G (p.P215A) variant outside of the RING domain in a Polish CMT family [51]. While the actual disease-causing mutation was identified in *RAB7A*, the authors hypothesize that the aggravated phenotype of one of the affected siblings was caused by the *LRSAM1* variant which was also found in the healthy father and sister of the two patients. Variants targeting this position are present in European and Asian populations with no homozygous carriers identified in > 141,456 individuals. Regrettably, no functional studies were performed and at the moment it is impossible to ascertain the impact of this variant.

Although no large GWAS studies identified *LRSAM1* as a potential modifier of neurodegenerative disease, all of these individual observations provide an attractive setting for studying LRSAM1 as a possible modifier of neurodegenerative diseases.

## Conclusions

CMT2P, associated with dominant and exceptionally recessive *LRSAM1* mutations, is usually characterized by slowly progressive axonal neuropathy mainly involving lower limbs, and adulthood onset. The pathological background is a primary neuronopathy affecting both motor and sensory neurons with length-dependent sensorimotor axonopathy. There is still a severe lack of understanding of the function of LRSAM1^WT^ and its perturbation by the CMT causing mutations, due to the scarcity of systematic functional studies performed. More research is required to determine its role in CMT and other neurodegenerative diseases with the aim to find therapeutic strategies to treat these incurable disorders.

## Supplementary Information

**Additional file 1.** Autopsy findings in LRSAM1.

## Abbreviations

CMT: Charcot–Marie–Tooth
ALS: Amyotrophic lateral sclerosis
SCA: Spinocerebellar ataxia
FTD: Frontotemporal dementia
PD: Parkinson’s disease
HSCR: Hirschsprung's disease
*LRSAM1*: Leucine rich repeat and sterile alpha motif containing 1
*MPZ*: Myelin protein zero
*PMP22*: Peripheral myelin protein 22
MBP: Myelin basic protein
GJB1: Gap junction beta-1
MFN2: Mitofusin 2
RING: Really interesting new gene
*TSG101*: Tumour susceptibility gene 101
UPS: Ubiquitin proteasome system
LRR: Leucine rich repeats
SAM: Sterile alpha motif
PTAP: Pro-Thr/Ser-Ala-Pro
CC: Coiled coil
ESCRT: Endosomal sorting complex required for transport
EGFR: Epidermal growth factor receptor
LITAF: LPS-induced TNF-activating factor
*FUS*: Fused in sarcoma
*G3BP1*: Ras GTPase-activating protein-binding protein 1
*HINT1*: Histidine Triad Nucleotide Binding Protein 1
*SH3TC2*: SH3 Domain and Tetratricopeptide Repeats 2
Htt: Huntingtin
*RAB7A*: Ras-related protein Rab-7a
EBV: Ebstein-Barr virus
CMTNS: CMT neuropathy score
NCV: Nerve conduction velocities
MRI: Magnetic resonance imaging
WES: Whole exome sequencing
PDB: Protein data bankEx
gnomAD: Genome Aggregation Database
KO: Knock out

## References

[1] Barreto LC, Oliveira FS, Nunes PS (2016). Epidemiologic study of Charcot–Marie–Tooth disease: a systematic review. Neuroepidemiology.

[2] Pareyson D, Saveri P, Pisciotta C (2017). New developments in Charcot–Marie–Tooth neuropathy and related diseases. Curr Opin Neurol.

[3] Dyck PJ, Lambert EH (1968). Lower motor and primary sensory neuron diseases with peroneal muscular atrophy. Arch Neurol.

[4] Thomas PK, Calne DB (1974). Motor nerve conduction velocity in peroneal muscular atrophy: evidence for genetic heterogeneity. J Neurol Neurosurg Psychiatry.

[5] Bradley WG, Madrid R, Davis CJ (1977). The peroneal muscular atrophy syndrome. Clinical genetic, electrophysiological and nerve biopsy studies. Part 3. Clinical, electrophysiological and pathological correlations. J Neurol Sci.

[6] Guernsey DL, Jiang H, Bedard K (2010). Mutation in the gene encoding ubiquitin ligase LRSAM1 in patients with Charcot–Marie–Tooth disease. PLoS Genet.

[7] Hakonen JE, Sorrentino V, Avagliano Trezza R (2017). LRSAM1-mediated ubiquitylation is disrupted in axonal Charcot–Marie–Tooth disease 2P. Hum Mol Genet.

[8] Hu B, Arpag S, Zuchner S, Li J (2016). A novel missense mutation of CMT2P alters transcription machinery. Ann Neurol.

[9] Nicolaou P, Cianchetti C, Minaidou A (2013). A novel LRSAM1 mutation is associated with autosomal dominant axonal Charcot–Marie–Tooth disease. Eur J Hum Genet.

[10] Peeters K, Palaima P, Pelayo-Negro AL (2016). Charcot–Marie–Tooth disease type 2G redefined by a novel mutation inLRSAM1. Ann Neurol.

[11] Weterman MA, Sorrentino V, Kasher PR (2012). A frameshift mutation in LRSAM1 is responsible for a dominant hereditary polyneuropathy. Hum Mol Genet.

[12] Engeholm M, Sekler J, Schöndorf DC (2014). A novel mutation in LRSAM1 causes axonal Charcot–Marie–Tooth disease with dominant inheritance. BMC Neurol.

[13] Lorefice L, Murru MR, Coghe G (2017). Charcot–Marie–Tooth disease: genetic subtypes in the Sardinian population. Neurol Sci.

[14] Dohrn MF, Glöckle N, Mulahasanovic L (2017). Frequent genes in rare diseases: panel-based next generation sequencing to disclose causal mutations in hereditary neuropathies. J Neurochem.

[15] Karczewski KJ, Francioli LC, Tiao G et al. Variation across 141,456 human exomes and genomes reveals the spectrum of loss-of-function intolerance across human protein-coding genes: Supplementary Information. 2019

[16] Peeters K, Chamova T, Jordanova A (2014). Clinical and genetic diversity of SMN1-negative proximal spinal muscular atrophies. Brain.

[17] Zhao G, Song J, Yang M, Song X, Liu X (2018). A novel mutation of LRSAM1 in a Chinese family with Charcot–Marie–Tooth disease. J Peripher Nerv Syst.

[18] Peretti A, Perie M, Vincent D (2019). LRSAM1 variants and founder effect in French families with ataxic form of Charcot–Marie–Tooth type 2. Eur J Hum Genet.

[19] Chiang CWK, Marcus JH, Sidore C (2018). Genomic history of the Sardinian population. Nat Genet.

[20] Shy ME, Blake J, Krajewski K (2005). Reliability and validity of the CMT neuropathy score as a measure of disability. Neurology.

[21] Berciano J, Combarros O, Figols J (1986). Hereditary motor and sensory neuropathy type II clinicopathological study of a family. Brain.

[22] Berciano J, Gallardo E, García A, Pelayo-Negro AL, Infante J, Combarros O (2011). New insights into the pathophysiology of pes cavus in Charcot–Marie–Tooth disease type 1A duplication. J Neurol.

[23] Aerts MB, Weterman MA, Quadri M (2016). A LRSAM1 mutation links Charcot–Marie–Tooth type 2 to Parkinson’s disease. Ann Clin Transl Neurol.

[24] Buchthal F, Behse F (1977). Peroneal muscular atrophy (PMA) and related disorders. I. Clinical manifestations as related to biopsy findings, nerve conduction and electromyography. Brain.

[25] Berciano J, García A, Gallardo E (2017). Intermediate Charcot–Marie–Tooth disease: an electrophysiological reappraisal and systematic review. J Neurol.

[26] McDonald B, Martin-Serrano J (2008). Regulation of Tsg101 expression by the steadiness box: a role of Tsg101-associated ligase. Mol Biol Cell.

[27] Deshaies RJ, Joazeiro CA (2009). RING domain E3 ubiquitin ligases. Annu Rev Biochem.

[28] Metzger MB, Pruneda JN, Klevit RE, Weissman AM (2014). RING-type E3 ligases: master manipulators of E2 ubiquitin-conjugating enzymes and ubiquitination. Biochim Biophys Acta.

[29] Liew C, Sun H, Hunter T, Day C (2010). RING domain dimerization is essential for RNF4 function. Biochem J.

[30] Bian W, Guo Y, Zhang Y, Li H (2017). The self-association and activity regulation of LRSAM1 E3 ligase. Biochem Biophys Res Commun.

[31] Amit I, Yakir L, Katz M (2004). Tal, a Tsg101-specific E3 ubiquitin ligase, regulates receptor endocytosis and retrovirus budding. Genes Dev.

[32] Huett A, Heath RJ, Begun J (2012). The LRR and RING domain protein LRSAM1 is an E3 ligase crucial for ubiquitin-dependent autophagy of intracellular Salmonella Typhimurium. Cell Host Microbe.

[33] Tofaris GK, Kim HT, Hourez R, Jung JW, Kim KP, Goldberg AL (2011). Ubiquitin ligase Nedd4 promotes alpha-synuclein degradation by the endosomal-lysosomal pathway. Proc Natl Acad Sci U S A.

[34] Li B, Su Y, Ryder J, Yan L, Na S, Ni B (2003). RIFLE: a novel ring zinc finger-leucine-rich repeat containing protein, regulates select cell adhesion molecules in PC12 cells. J Cell Biochem.

[35] Stuchell MD, Garrus JE, Müller B (2004). The human endosomal sorting complex required for transport (ESCRT-I) and its role in HIV-1 budding. J Biol Chem.

[36] Lee SM, Chin LS, Li L (2017). Dysregulation of ErbB receptor trafficking and signaling in demyelinating Charcot–Marie–Tooth disease. Mol Neurobiol.

[37] Qin W, Wunderley L, Barrett AL, High S, Woodman PG (2016). The Charcot Marie Tooth disease protein LITAF is a zinc-binding monotopic membrane protein. Biochem J.

[38] Qiu H, Lee S, Shang Y (2014). ALS-associated mutation FUS-R521C causes DNA damage and RNA splicing defects. J Clin Invest.

[39] Aulas A, Caron G, Gkogkas CG (2015). G3BP1 promotes stress-induced RNA granule interactions to preserve polyadenylated mRNA. J Cell Biol.

[40] Bogdanik LP, Sleigh JN, Tian C (2013). Loss of the E3 ubiquitin ligase LRSAM1 sensitizes peripheral axons to degeneration in a mouse model of Charcot–Marie–Tooth disease. Dis Model Mech.

[41] Sievers F, Wilm A, Dineen D (2014). Fast, scalable generation of high-quality protein multiple sequence alignments using clustal omega. Molecular Systems Biology.

[42] Seburn KL, Morelli KH, Jordanova A, Burgess RW (2014). Lack of neuropathy-related phenotypes in hint1 knockout mice. J Neuropathol Exp Neurol.

[43] Ibañez-Juliá MJ, Berzero G, Reyes-Botero G (2018). Antineoplastic agents exacerbating Charcot Marie Tooth disease: red flags to avoid permanent disability. Acta Oncol.

[44] Kim SH, Shanware NP, Bowler MJ, Tibbetts RS (2010). Amyotrophic lateral sclerosis-associated proteins TDP-43 and FUS/TLS function in a common biochemical complex to co-regulate HDAC6 mRNA. J Biol Chem.

[45] Gamage KK, Cheng I, Park RE (2017). Death receptor 6 promotes Wallerian degeneration in peripheral axons. Curr Biol.

[46] Zhang Y, He X, Meng X (2017). Regulation of glutamate transporter trafficking by Nedd4-2 in a Parkinson’s disease model. Cell Death Disease.

[47] Tang B, Seredenina T, Coppola G (2011). Gene expression profiling of R6/2 transgenic mice with different CAG repeat lengths reveals genes associated with disease onset and progression in Huntington’s disease. Neurobiol Dis.

[48] Chen G, Du C, Shen Z (2018). MicroRNA-939 inhibits cell proliferation via targeting LRSAM1 in Hirschsprung’s disease. Aging (Albany NY).

[49] Nolan M, Talbot K, Ansorge O (2016). Pathogenesis of FUS-associated ALS and FTD: insights from rodent models. Acta Neuropathol Commun.

[50] Neumann M, Sampathu DM, Kwong LK (2006). Ubiquitinated TDP-43 in frontotemporal lobar degeneration and amyotrophic lateral sclerosis. Science.

[51] Peddareddygari LR, Oberoi K, Vellore JR, Grewal RP (2016). Factors Affecting Phenotype Variability in a Family with CMT2B: Gender and LRSAM1 Genotype. Case Rep Neurol.

[52] Mortreux L, Bacquet J, Boyer A, Alazard E, Bellance R, Giguet-Valard AG, Cerino M, Krahn M, Audic F, Chabrol B, Laugel V, Desvignes JP, Béroud C, Nguyen K, Verschueren A, Lévy N, Attarian S, Delague V, Missirian C, Bonello-Palot N (2020). Identification of novel pathogenic copy number variations in Charcot–Marie–Tooth disease. J Hum Genet.

