# Supplementary material for: LRSAM1 and the RING domain: Charcot–Marie–Tooth disease and beyond
Source: Orphanet J Rare Dis. 2021 Feb 10;16:74. doi: 10.1186/s13023-020-01654-8 (PMC7874611; doi:10.1186/s13023-020-01654-8)
Supplement: Supplementary file 1 — Additional file 1. Autopsy findings in LRSAM1. [file 13023_2020_1654_MOESM1_ESM.docx]

**Palaima et al.**

**Additional information**

**Supplementary figure 1. Necropsy findings.**


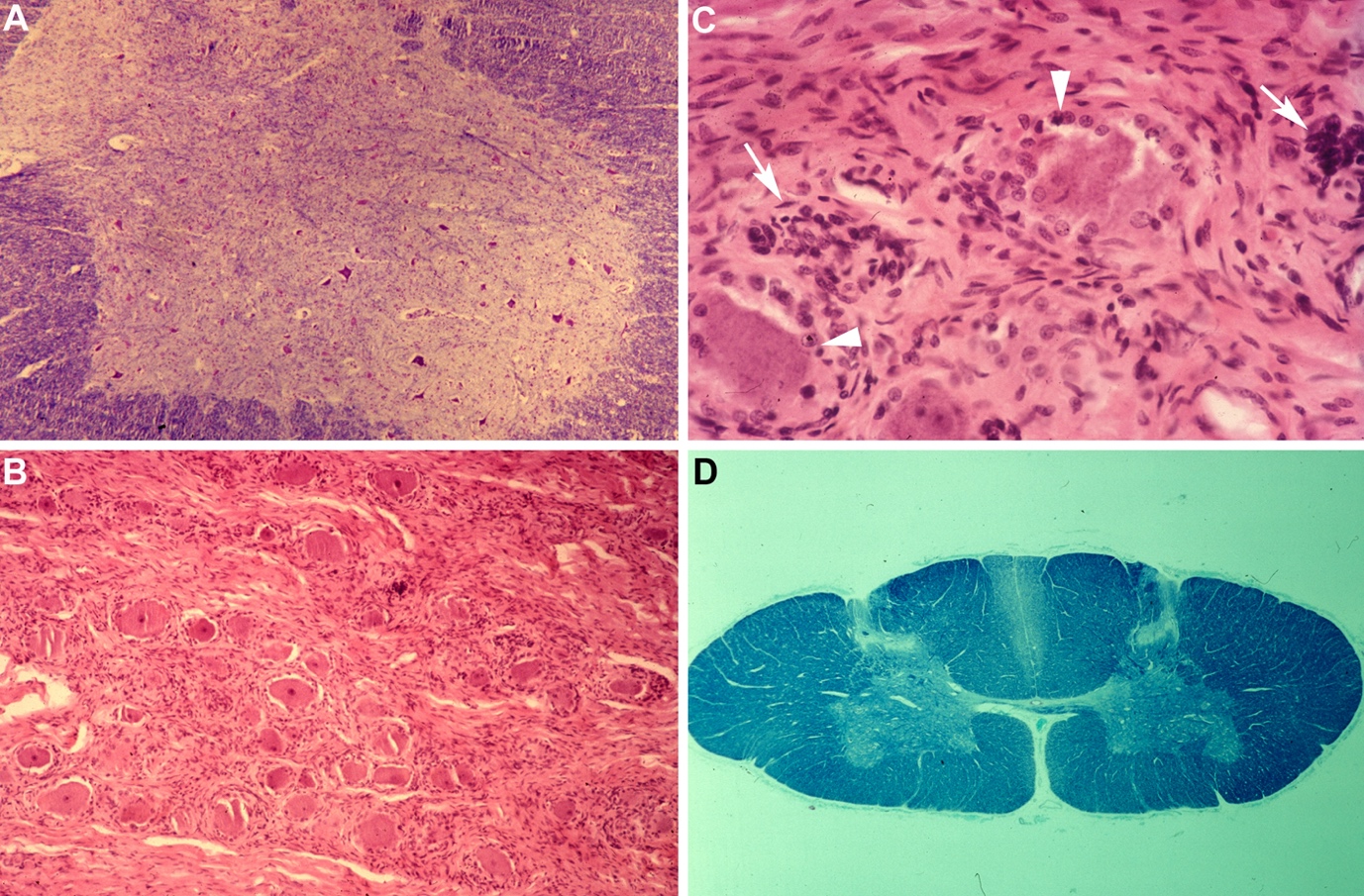


Propositus died at age 32 years (Case III-7 in (21) and Case III-4 in (10)). (***A***) Transverse section of the spinal cord at S1 segmental level showing loss of motor neurons in anterior horn accompanied by gliosis (Klüver-Barrera, x25 before reduction). (***B***) Lumbar posterior root ganglion showing loss of neurons and an increase in fibrous tissue; higher power (***C***) showing degenerated neuronal cell bodies with proliferation of capsule cells (arrowheads) and residual nodules of Nageotte (arrows) (H&E, x80 and x160 before reduction). (***D***) Transverse section of the spinal cord at C5 segmental level showing bilateral demyelination of fasciculus gracillis (Klüver-Barrera, x10 before reduction); Holzer staining demonstrated that demyelination was accompanied by isomorphic fibrillary gliosis (not shown). Adapted from (21)*.*
